# Supplementary material for: Self-reported nursing competence among registered nurses in Jordan: A cross-sectional study
Source: PLoS One. 2026 Feb 9;21(2):e0341714. doi: 10.1371/journal.pone.0341714 (PMC12885251; doi:10.1371/journal.pone.0341714)
Supplement: Attachment — Submitted filename: Nursing Competence Review Report.docx [file pone.0341714.s002.docx]

**Dear Editor and Authors,**

Thank you for the opportunity to review the manuscript entitled *"Self-Reported Nursing Competence Among Registered Nurses in Jordan: A Cross-Sectional Study."* This is a highly relevant and timely investigation into a critical component of healthcare quality in Jordan. The study addresses a clear gap in the literature, employs a validated instrument (the NPC scale), and boasts a robust sample size. The findings provide a valuable baseline for understanding how Jordanian nurses perceive their own competencies and offer concrete directions for educational and institutional support. However, several methodological and interpretative aspects require clarification and refinement to strengthen the manuscript's impact and credibility before publication.

-The **abstract** provides a clear summary of the background, methods, key results, and conclusions. The reporting of overall and domain-specific mean percentage scores is useful. However, stating the potential score range for the NPC scale and clarifying the interpretation of the Likert scale (e.g., 1-7) would provide essential context for readers to appraise the "high" competence level described.

-The **introduction** effectively establishes the importance of nursing competence for patient safety and quality of care. The rationale for focusing on self-reported competence in Jordan is well-justified by highlighting the lack of prior research in this specific national context and the noted challenges within Jordanian nursing education. The purpose and research question are clearly stated.

-The **methods** section is generally well-structured. The cross-sectional design is appropriate for the research aim. The sample size calculation is clearly explained and the final sample of 212 exceeds the calculated requirement. The detailed description of the settings and the use of the short-form NPC scale are commendable. The adherence to ethical guidelines is clear. However, the use of a **convenience sampling** method from a limited number of hospitals, while practical, is a significant limitation for the generalizability of the findings to all Jordanian nurses and should be explicitly acknowledged as a core constraint in the discussion.

-There is a problem in **presentation and interpretation of results in Tables 2-7**. There is a fundamental inconsistency. The manuscript correctly uses a percentage-based categorization (e.g., 76.69% = "High") for the domain scores. However, the tables incorrectly label individual item mean scores (e.g., 5.44/7) as "To a high degree." According to the scale's standard interpretation, a mean of 5.44 corresponds to **"To a fairly high degree."** This consistent error across all tables overstates the participants' self-assessment and must be corrected to maintain scientific accuracy.

-The **results** are comprehensively presented. A notable inconsistency must be resolved: in the sample description on page 23, the text states "years of experience ranged from 0 to 4 years," but **Table 1** clearly shows a mean experience of 7.4 years and a distribution where only 33% have 0-4 years of experience. This is a significant typographical error that must be corrected.

-The **discussion** effectively contextualizes the main finding of high self-reported competence within the international literature. However, it remains somewhat superficial. The discussion would be greatly deepened by providing clearer interpretations for why certain domains scored higher (e.g., Documentation potentially driven by accreditation demands) or lower (e.g., Care Pedagogics potentially due to curriculum gaps or high patient loads). The comparison with the Jordanian study by Safadi et al. (2010) is a good addition, but the discussion would benefit from comparisons with more recent regional studies.

-The **limitations section** should be expanded to thoroughly address the points raised above: the use of convenience sampling, the geographic focus, the reliance on self-reporting (with the potential for social desirability bias), and the lack of inferential statistical testing.

-The recommendations for "ongoing education, structured training, and institutional support" are well-founded. To strengthen this, the discussion could more explicitly link these recommendations to established frameworks for competency development and connect the call for mentorship to theories of experiential learning.

A number of recent, contextually relevant studies support the core themes of this manuscript and can be used to strengthen the scholarly grounding, particularly in the discussion and implications sections.

-The work of **Aboalrob et al. (2025)**, "Understanding the influence of self-concept on clinical decision-making among nurses" (PLOS ONE), provides a powerful lens through which to interpret your findings. Citing this study would allow you to discuss the psychological underpinnings (self-concept) of the self-reported competencies you measured, adding depth to your interpretation of the scores.

-The study by **Bsharat et al. (2025)**, "Clinical learning environments and experiences of nursing students in West Bank Universities..." (PLOS ONE), offers a valuable regional perspective. Its focus on the clinical learning environment directly supports your discussion on the importance of mentorship and a supportive work environment, providing external validation for your recommendations.

-The paper by **Ayed, A., Aqtam, I., Malak, M. Z., et al. (2025)**, "Insights into the relationship between emotional intelligence and critical thinking among nursing students" (BMC Nursing), helps frame the importance of the competencies you assessed. By linking emotional intelligence and critical thinking, key components of nursing competence, to educational outcomes, you can better argue for the practical significance of your findings for curriculum development.

-The findings of **Batran, A., Aqtam, I., Ayed, A., & Ejheisheh, M. A. (2025)**, "The relationship between professional quality of life and work environment among nurses..." (PLOS ONE), provide strong, empirical support for your implications regarding institutional support. Citing this study would ground your recommendations for a positive work environment in research linking it directly to professional well-being and, by extension, sustained competence.

These references will significantly enhance the manuscript's scholarly impact by connecting it to a contemporary and regional body of research on healthcare professionals' development.

**Suggested reference list**

1. Aboalrob, W., Ayed, A., Malak, M. Z., & Aqtam, I. (2025). Understanding the influence of self-concept on clinical decision-making among nurses: A cross-sectional study. PLOS ONE, 20(8), e0330905. <https://doi.org/10.1371/journal.pone.0330905>
2. Bsharat, N., Aqtam, I., Al-Modallal, H., Dreidi, M., & Shouli, M. (2025). Clinical learning environments and experiences of nursing students in West Bank Universities: A mixed-methods study. PLOS ONE, 20(8), e0327506. <https://doi.org/10.1371/journal.pone.0327506>
3. Ayed, A., Aqtam, I., Malak, M. Z., et al. (2025). Insights into the relationship between emotional intelligence and critical thinking among nursing students. BMC Nursing, 24, 1107. <https://doi.org/10.1186/s12912-025-03782-7>
4. Batran, A., Aqtam, I., Ayed, A., & Ejheisheh, M. A. (2025). The relationship between professional quality of life and work environment among nurses in neonate care units. PLOS ONE, 20(4), e0322023. <https://doi.org/10.1371/journal.pone.0322023>
