# Supplementary material for: Self-reported nursing competence among registered nurses in Jordan: A cross-sectional study
Source: PLoS One. 2026 Feb 9;21(2):e0341714. doi: 10.1371/journal.pone.0341714 (PMC12885251; doi:10.1371/journal.pone.0341714)
Supplement: Attachment — Submitted filename: Response to Reviewers.docx [file pone.0341714.s004.docx]

**Response to Reviewers**

**Editor’s comments**

When submitting your revision, we need you to address these additional requirements.

1. Please ensure that your manuscript meets PLOS ONE's style requirements, including those for file naming. The PLOS ONE style templates can be found at

<https://journals.plos.org/plosone/s/file?id=wjVg/PLOSOne_formatting_sample_main_body.pdf> and 
<https://journals.plos.org/plosone/s/file?id=ba62/PLOSOne_formatting_sample_title_authors_affiliations.pdf>

Response: Note taken and changes applied as requested.

2. Please include a complete copy of PLOS’ questionnaire on inclusivity in global research in your revised manuscript. Our policy for research in this area aims to improve transparency in the reporting of research performed outside of researchers’ own country or community. The policy applies to researchers who have travelled to a different country to conduct research, research with Indigenous populations or their lands, and research on cultural artefacts. The questionnaire can also be requested at the journal’s discretion for any other submissions, even if these conditions are not met.

Please find more information on the policy and a link to download a blank copy of the questionnaire here: <https://journals.plos.org/plosone/s/best-practices-in-research-reporting>.

Please upload a completed version of your questionnaire as Supporting Information when you resubmit your manuscript.

Response: The form was completed as requested.

3. We noticed you have some minor occurrence of overlapping text with the following previous publication(s), which needs to be addressed:

<https://www.sciedupress.com/journal/index.php/jnep/article/viewFile/22609/14362>
<https://search.informit.org/doi/abs/10.3316/informit.223994226712264>

In your revision ensure you cite all your sources (including your own works), and quote or rephrase any duplicated text outside the methods section. Further consideration is dependent on these concerns being addressed.

Response: We reviewed the manuscript in relation to the cited publications and identified minor similarities limited to the background and literature review, primarily involving conceptual definitions of nursing competence, the rationale for self-assessment, and general descriptions of the NPC scale. All overlapping text outside the Methods section has been rephrased, and all relevant sources, including our prior publications, have been appropriately cited. The revised manuscript represents a distinct study with a different sample, setting, and research focus, and we believe these revisions adequately address the concerns regarding overlap and ensure transparency and scholarly integrity.

4. We note that the grant information you provided in the ‘Funding Information’ and ‘Financial Disclosure’ sections do not match.

When you resubmit, please ensure that you provide the correct grant numbers for the awards you received for your study in the ‘Funding Information’ section.

Response: I do not have a grant number, Georgetown University Medical Center, supported the publication of this paper financially by having the agreement with PLOS One, but they did not provide other financial support for the study implementation, so I do not have a grant number.

5. Thank you for stating the following financial disclosure:

“Funding support is in part from Georgetown University Medical Center.“

Please state what role the funders took in the study.  If the funders had no role, please state: "The funders had no role in study design, data collection and analysis, decision to publish, or preparation of the manuscript." If this statement is not correct you must amend it as needed.

Please include this amended Role of Funder statement in your cover letter; we will change the online submission form on your behalf.

Response: This was changed as requested

6. Thank you for stating in your Funding Statement:

“Funding support is in part from Georgetown University Medical Center.“

Please provide an amended statement that declares *all* the funding or sources of support (whether external or internal to your organization) received during this study, as detailed online in our guide for authors at <http://journals.plos.org/plosone/s/submit-now>.  Please also include the statement “There was no additional external funding received for this study.” in your updated Funding Statement.

Please include your amended Funding Statement within your cover letter. We will change the online submission form on your behalf.

Response: The statement was added as requested.

7. Please note that funding information should not appear in the Acknowledgments section or other areas of your manuscript. We will only publish funding information present in the Funding Statement section of the online submission form. Please remove any funding-related text from the manuscript.

Response: Funding information was not added to the manuscript.

8. We note that your Data Availability Statement is currently as follows:

“All relevant data are within the manuscript and its Supporting Information files.”

Please confirm at this time whether or not your submission contains all raw data required to replicate the results of your study. Authors must share the “minimal data set” for their submission. PLOS defines the minimal data set to consist of the data required to replicate all study findings reported in the article, as well as related metadata and methods (<https://journals.plos.org/plosone/s/data-availability#loc-minimal-data-set-definition>).

For example, authors should submit the following data:

- The values behind the means, standard deviations and other measures reported;
- The values used to build graphs;
- The points extracted from images for analysis.

Authors do not need to submit their entire data set if only a portion of the data was used in the reported study.

If your submission does not contain these data, please either upload them as Supporting Information files or deposit them to a stable, public repository and provide us with the relevant URLs, DOIs, or accession numbers. For a list of recommended repositories, please see <https://journals.plos.org/plosone/s/recommended-repositories>.

If there are ethical or legal restrictions on sharing a de-identified data set, please explain them in detail (e.g., data contain potentially sensitive information, data are owned by a third-party organization, etc.) and who has imposed them (e.g., an ethics committee). Please also provide contact information for a data access committee, ethics committee, or other institutional body to which data requests may be sent. If data are owned by a third party, please indicate how others may request data access.

Response: Data would be available from the PI upon reasonable request.

9. Your ethics statement should only appear in the Methods section of your manuscript. If your ethics statement is written in any section besides the Methods, please delete it from any other section.

Response: The ethics statement only appear in the Methods section of your manuscript.

10. We note you have included a table to which you do not refer in the text of your manuscript. Please ensure that you refer to Table 1 in your text; if accepted, production will need this reference to link the reader to the Table.

Response: Table (1) was added as requested.

11. If the reviewer comments include a recommendation to cite specific previously published works, please review and evaluate these publications to determine whether they are relevant and should be cited. There is no requirement to cite these works unless the editor has indicated otherwise.

Response: Note taken.

12. Please review your reference list to ensure that it is complete and correct. If you have cited papers that have been retracted, please include the rationale for doing so in the manuscript text, or remove these references and replace them with relevant current references. Any changes to the reference list should be mentioned in the rebuttal letter that accompanies your revised manuscript. If you need to cite a retracted article, indicate the article’s retracted status in the References list and also include a citation and full reference for the retraction notice.

Response: Note taken.

| **Revision** | **Reviewers' Comments** |
| --- | --- |
| Thank you for this positive and constructive feedback. | Reviewer #1: This study provides valuable insight into the self-perceived competence of registered nurses in Jordan, addressing an important gap in the national nursing literature. the paper contributes meaningfully to efforts aimed at enhancing nursing quality and patient safety in Jordan. |
| Thank you for this positive and constructive feedback. | Reviewer #2: This manuscript presents a valuable and timely investigation into self-reported nursing competence in Jordan. The study addresses a clear gap in the literature, utilizes a validated instrument, and has a robust sample size. The findings are relevant for nursing education and practice in the region. However, several issues require attention to enhance the manuscript's scientific rigor, clarity, and impact before it is suitable for publication. |
| The sentence was changed as requested. See page 3  This keyword was added as requested. See page 4.  Thank you for this important suggestion. The Ethics Considerations section has been revised to explicitly state that written informed consent was obtained from all participants and that participants were fully informed of their right to withdraw from the study at any time without consequences. See page 13.  The description was paraphrased and clarified. See page 15.  Thank you for this comment. Inferential statistical analyses were not conducted for this section of the data.  The table’s formatting was revised as requested.  This section was further clarified as requested. See page 18.  This limitation was further clarified as requested, see page 20.  Proofreading was done as requested. | Reviewer #3: Abstract Clarity: While the abstract provides a good overview, consider rephrasing the sentence "Despite nurses' essential contributions, limited research has examined self-reported nursing competence (SRNC) in Jordan" to emphasize the gap in research more directly. For example, "Despite the critical role of nurses, research on self-reported nursing competence (SRNC) in Jordan remains limited."  Keyword Refinement: Consider adding "Cross-Sectional Study" to the keywords to improve searchability.  Ethical Statement: Ensure the ethics statement includes a mention of informed consent and that participants were aware of their right to withdraw from the study at any time.  Sample Description: Clarify the phrase, "years of experience ranged from 0 to 4 years." Does this mean the range was very limited, or should this be a higher number? Furthermore, specify whether the experience is in nursing.  Statistical Analysis: Briefly mention any specific statistical tests used beyond descriptive statistics (e.g., t-tests, ANOVA) if applicable.  Table Formatting: Ensure all tables are clearly labelled and that column headings are concise and easily understood. Check for consistency in decimal places.  Discussion – Comparison to Other Studies: When comparing findings to Safadi et al. (43), elaborate further on the differences in perspectives (self-assessment vs. manager assessment) and how these distinctions might explain any discrepancies.  Limitations – Response Bias: Elaborate on the possible consequences of response bias in the self-assessment questionnaires. How might this specifically affect the interpretation of the results?  Typos and Grammar: Proofread carefully for any remaining typos or grammatical errors. |
| The context information was added as requested, see pages 5 and 6.  The question was revised as requested, see page 7.  This NPC description was added as requested, see page 11.  Thank you for your feedback.  These examples are actually added to the result section.  This was added as requested, see pages 21 and 22.  I used RefWorks tool for references, and I am happy to modify them as needed.  This statement was added as requested, see page 20 | Reviewer #4: Specific Comments and Suggestions Introduction–Jordan Healthcare System Context: Specific Comment: The introduction would benefit from a brief overview of the Jordanian healthcare system. Consider adding a sentence or two about its structure (e.g., public vs. private provision), key challenges (e.g., resource constraints, access disparities), and any recent reforms or initiatives. This will help readers understand the context in which nursing competence is being assessed. Suggestion: Consult sources like the World Health Organization (WHO) or recent publications on healthcare in Jordan to gather this contextual information.  Research Question Refinement: Specific Comment: The current research question is broad. Rephrasing it to be more specific will improve the focus of the study. Suggestion: Instead of "What is the level of self-reported professional nursing competence among registered nurses in Jordan?" try: "What is the self-reported level of professional nursing competence among registered nurses in Jordan across different domains of practice, as measured by the Nursing Professional Competence (NPC) scale?" This clarifies the measurement instrument and the scope of the assessment.  Instrumentation - NPC Scale Description: Specific Comment: The Methods section lacks detail about the domains assessed by the NPC scale. Readers need to know what specific aspects of nursing competence were evaluated. Suggestion: Add a sentence or two describing the six competence areas measured by the NPC scale (Nursing Care, value-based care, etc.). For example: "The NPC scale assesses six key areas of nursing competence: Nursing Care, Value-Based Care, Medical and Technical Care, Care Pedagogics, Documentation and Administration, and Development, Leadership, and Organization of Care."  Results - Examples of High and Low-Scoring Items: Specific Comment: It's difficult to interpret the meaning of the high and low overall scores without knowing which specific items contributed most to those scores. Suggestion: In the Results section, when discussing the high- and low-scoring areas, provide a few examples of the specific items from the NPC scale that received the highest and lowest ratings. For example: "The highest-rated item was 'Document the patient's physical condition', while the lowest-rated item was 'Address the patient's psychological condition.'"  Conclusion – Practical Implications: Specific Comment: The conclusion should offer more concrete recommendations based on the study's findings. What specific actions can be taken to improve nursing competence in Jordan? Suggestion: Expand the conclusion to include actionable recommendations for nursing education and practice. For example: "Based on these findings, nursing education programs in Jordan should consider strengthening curricula in areas such as [specific area needing improvement], while healthcare institutions should focus on providing ongoing professional development opportunities in [specific area needing improvement]."  References – Consistent Formatting: Specific Comment: The reference list may have inconsistencies in formatting. Suggestion: Carefully review all references to ensure they adhere to the PLOS ONE style guidelines. Pay attention to journal abbreviations, capitalization, italics, and the order of elements.  Limitations - Generalizability: Specific Comment: The study's generalizability may be limited by the sampling method. Note in the Limitations section that the findings may not represent all Jordanian nurses due to the convenience sampling method and the specific hospitals included in the study. For example: "The use of convenience sampling limits the generalizability of the findings to all nurses in Jordan. Future research should employ random sampling methods to obtain a more representative sample." |
| Thank you for this positive and constructive feedback.  The abstract was revised as requested, see page 3**.**  Thank you for this positive and constructive feedback.  This limitation was further discussed in the limitation section, see page 20.  Thank you for your feedback, the result section was revised as requested. See pages 14-16.  This was revised as requested, see page 15.  The section was revised as requested, see page 17.  This section was modified as requested, see pages 20 and 21.  This section was revised as requested. See page 20.  Thank you for your valuable feedback, three of the suggested studies were added to the introduction, discussion, and the implication sections of this paper. | **Dear Editor and Authors,**  Thank you for the opportunity to review the manuscript entitled *"Self-Reported Nursing Competence Among Registered Nurses in Jordan: A Cross-Sectional Study."* This is a highly relevant and timely investigation into a critical component of healthcare quality in Jordan. The study addresses a clear gap in the literature, employs a validated instrument (the NPC scale), and boasts a robust sample size. The findings provide a valuable baseline for understanding how Jordanian nurses perceive their own competencies and offer concrete directions for educational and institutional support. However, several methodological and interpretative aspects require clarification and refinement to strengthen the manuscript's impact and credibility before publication.  -The **abstract** provides a clear summary of the background, methods, key results, and conclusions. The reporting of overall and domain-specific mean percentage scores is useful. However, stating the potential score range for the NPC scale and clarifying the interpretation of the Likert scale (e.g., 1-7) would provide essential context for readers to appraise the "high" competence level described.  -The **introduction** effectively establishes the importance of nursing competence for patient safety and quality of care. The rationale for focusing on self-reported competence in Jordan is well-justified by highlighting the lack of prior research in this specific national context and the noted challenges within Jordanian nursing education. The purpose and research question are clearly stated.  -The **methods** section is generally well-structured. The cross-sectional design is appropriate for the research aim. The sample size calculation is clearly explained and the final sample of 212 exceeds the calculated requirement. The detailed description of the settings and the use of the short-form NPC scale are commendable. The adherence to ethical guidelines is clear. However, the use of a **convenience sampling** method from a limited number of hospitals, while practical, is a significant limitation for the generalizability of the findings to all Jordanian nurses and should be explicitly acknowledged as a core constraint in the discussion.  -There is a problem in **presentation and interpretation of results in Tables 2-7**. There is a fundamental inconsistency. The manuscript correctly uses a percentage-based categorization (e.g., 76.69% = "High") for the domain scores. However, the tables incorrectly label individual item mean scores (e.g., 5.44/7) as "To a high degree." According to the scale's standard interpretation, a mean of 5.44 corresponds to **"To a fairly high degree."** This consistent error across all tables overstates the participants' self-assessment and must be corrected to maintain scientific accuracy.  -The **results** are comprehensively presented. A notable inconsistency must be resolved: in the sample description on page 23, the text states "years of experience ranged from 0 to 4 years," but **Table 1** clearly shows a mean experience of 7.4 years and a distribution where only 33% have 0-4 years of experience. This is a significant typographical error that must be corrected.  -The **discussion** effectively contextualizes the main finding of high self-reported competence within the international literature. However, it remains somewhat superficial. The discussion would be greatly deepened by providing clearer interpretations for why certain domains scored higher (e.g., Documentation potentially driven by accreditation demands) or lower (e.g., Care Pedagogics potentially due to curriculum gaps or high patient loads). The comparison with the Jordanian study by Safadi et al. (2010) is a good addition, but the discussion would benefit from comparisons with more recent regional studies.  -The **limitations section** should be expanded to thoroughly address the points raised above: the use of convenience sampling, the geographic focus, the reliance on self-reporting (with the potential for social desirability bias), and the lack of inferential statistical testing.  -The recommendations for "ongoing education, structured training, and institutional support" are well-founded. To strengthen this, the discussion could more explicitly link these recommendations to established frameworks for competency development and connect the call for mentorship to theories of experiential learning.  A number of recent, contextually relevant studies support the core themes of this manuscript and can be used to strengthen the scholarly grounding, particularly in the discussion and implications sections.  -The work of **Aboalrob et al. (2025)**, "Understanding the influence of self-concept on clinical decision-making among nurses" (PLOS ONE), provides a powerful lens through which to interpret your findings. Citing this study would allow you to discuss the psychological underpinnings (self-concept) of the self-reported competencies you measured, adding depth to your interpretation of the scores.  -The study by **Bsharat et al. (2025)**, "Clinical learning environments and experiences of nursing students in West Bank Universities..." (PLOS ONE), offers a valuable regional perspective. Its focus on the clinical learning environment directly supports your discussion on the importance of mentorship and a supportive work environment, providing external validation for your recommendations.  -The paper by **Ayed, A., Aqtam, I., Malak, M. Z., et al. (2025)**, "Insights into the relationship between emotional intelligence and critical thinking among nursing students" (BMC Nursing), helps frame the importance of the competencies you assessed. By linking emotional intelligence and critical thinking, key components of nursing competence, to educational outcomes, you can better argue for the practical significance of your findings for curriculum development.  -The findings of **Batran, A., Aqtam, I., Ayed, A., & Ejheisheh, M. A. (2025)**, "The relationship between professional quality of life and work environment among nurses..." (PLOS ONE), provide strong, empirical support for your implications regarding institutional support. Citing this study would ground your recommendations for a positive work environment in research linking it directly to professional well-being and, by extension, sustained competence.  These references will significantly enhance the manuscript's scholarly impact by connecting it to a contemporary and regional body of research on healthcare professionals' development.  **Suggested reference list**   1. Aboalrob, W., Ayed, A., Malak, M. Z., & Aqtam, I. (2025). Understanding the influence of self-concept on clinical decision-making among nurses: A cross-sectional study. PLOS ONE, 20(8), e0330905. <https://doi.org/10.1371/journal.pone.0330905> 2. Bsharat, N., Aqtam, I., Al-Modallal, H., Dreidi, M., & Shouli, M. (2025). Clinical learning environments and experiences of nursing students in West Bank Universities: A mixed-methods study. PLOS ONE, 20(8), e0327506. <https://doi.org/10.1371/journal.pone.0327506> 3. Ayed, A., Aqtam, I., Malak, M. Z., et al. (2025). Insights into the relationship between emotional intelligence and critical thinking among nursing students. BMC Nursing, 24, 1107. <https://doi.org/10.1186/s12912-025-03782-7> 4. Batran, A., Aqtam, I., Ayed, A., & Ejheisheh, M. A. (2025). The relationship between professional quality of life and work environment among nurses in neonate care units. PLOS ONE, 20(4), e0322023. <https://doi.org/10.1371/journal.pone.0322023> |
